# Before-after, control-impact analysis of evidence for the impacts of water level on Walleye, Northern Pike and Yellow Perch in lakes of the Rainy-Namakan complex (MN, USA and ON, CA)

**DOI:** 10.1371/journal.pone.0198612

**Published:** 2018-06-07

**Authors:** James H. Larson, Ryan P. Maki, Benjamin A. Vondra, Kevin E. Peterson

**Affiliations:** 1 U.S. Geological Survey, Upper Midwest Environmental Sciences Center, La Crosse, WI, United States of America; 2 National Park Service, Voyageurs National Park, Grand Rapids, MN, United States of America; 3 Minnesota Department of Natural Resources, International Falls, MN, United States of America; University of Waikato, NEW ZEALAND

## Abstract

Water level (WL) fluctuations in lakes influence many aspects of ecosystem processes. Concern about the potential impact of WL fluctuations on fisheries was one of the factors that motivated the decision in 2000 to alter the management of WL in the Rainy-Namakan reservoir complex (on the border between the U.S. state of Minnesota and the Canadian province of Ontario). We used a Before-After, Control-Impact (BACI) framework to identify potential impacts of the change in WL management to Walleye, Northern Pike and Yellow Perch catch per unit effort (CPUE). The CPUE of these species from 1990–1999 and from 2005–2014 were compared in four impact lakes (Lake Kabetogama, Namakan Lake, Rainy Lake and Sand Point Lake) and two control lakes (Lake of the Woods and Lake Vermilion) using a simple Bayesian model. Changes in fish CPUE in the impact lakes were often similar to changes that occurred in at least one control lake. The only change that was not similar to changes in control lakes was an increase of Yellow Perch in Lake Kabetogama. The two control lakes often differed substantially from each other, such that if only one had been available our conclusions about the role of WL management on fisheries would be very different. In general, identifying cause-and-effect relationships in observational field data is very difficult, and the BACI analysis used here does not specify a causative mechanism, so co-occurring environmental and management changes may obscure the effect of WL management.

## Introduction

Humans have frequently altered the water-level fluctuations in natural and artificial water bodies, and these water-level fluctuations may influence fisheries through a wide variety of mechanisms [1]. For Walleye and Northern Pike, the suitability and accessibility of spawning and nursery areas may be impacted by water level regimes [2,3]. For generalist species such as Yellow Perch (*Perca flavescens*), higher water levels may simply indicate an increase in available habitat [4]. Water levels may also impact fish species indirectly by altering the composition or productivity of primary producers [5–7] or the invertebrate community [8–10].

Concern about the potential impact of water level fluctuations on fisheries was an important factor behind the decision in 2000 to alter the management of water levels in the Rainy-Namakan Reservoir complex [2,11]. The Rainy-Namakan Reservoir complex includes several large lakes on the border between the U.S. state of Minnesota and the Canadian province of Ontario [12]. Anthropogenic alterations to water level fluctuations had been implicated as a factor suppressing populations of Walleye (*Sander vitreus)* and Northern Pike (*Esox lucius*) in these lakes [2,3,13,14]. In particular, low water levels in early spring are thought to reduce spawning habitat for these two species [2,15]. Water-level management is overseen in the Rainy/Namakan Reservoir complex by the International Joint Commission (IJC), which changed the rules governing water-levels in these lakes in 2000. In the 2000 Rule Curves, WL management for the Namakan Reservoir was changed to a more natural hydrologic regime by decreasing the magnitude of the winter drawdown on Namakan Reservoir by approximately 1 m [11]. This was intended to reduce potential negative effects on the production of Walleye, Northern Pike and other ecosystem processes. The 2000 Rule Curves for Rainy Lake brought about a subtle change in water levels than the change on Namakan Reservoir. The changes the 2000 Rule Curves established for Rainy Lake were a slightly wider band of allowable water levels during the spring refill period, and a slow drawdown through late summer and fall [11]. These subtle changes were expected to have some ecosystem benefits, including improvements in Walleye spawning habitat and Northern Pike nursery habitat.

There are several ways to evaluate the effectiveness of this new water level strategy. For relationships that are clearly defined, mechanistic models can be used to identify whether changes in water level management could result in the desired changes in fisheries [15]. However, water level management influences a wide array of ecosystem properties, and it is the net effect of these influences that likely impacts fisheries over the long term. Here we use the before-after, control-impact (BACI) framework to investigate whether changes in an index of fish abundance are likely associated with implementation of the 2000 Rule Curves [16]. In the BACI approach, an impacted system is compared to a control system before and after a treatment is initiated, without specifying a mechanistic pathway. Our objective was to estimate whether or not the 2000 Rule Curves are likely associated with changes in an index of abundance for Walleye, Northern Pike and Yellow Perch.

## Methods

### Ethics statement

All data used in this analysis was collected using protocols approved by the Minnesota Department of Natural Resources.

### Study lakes

**Fig 1:**
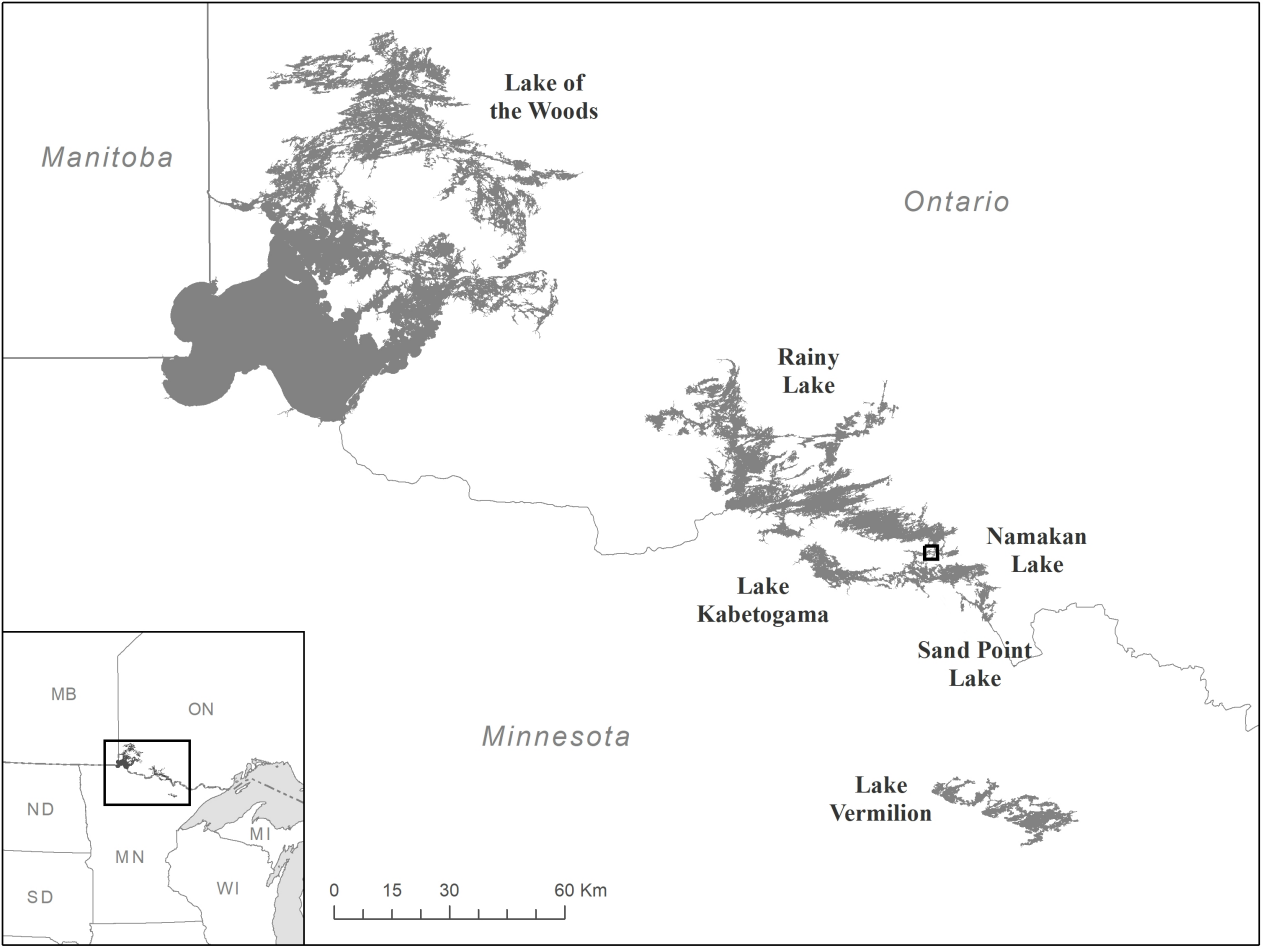
Map showing study lakes. The box identifies the area where dams regulate water movements from Namakan Lake into Rainy Lake. These dams also determine water levels in Lake Kabetogama and Sand Point Lake, which both flow into Namakan Lake. Lake Vermilion is upstream of the Namakan complex and its water levels are not influenced by water level management at these dams. Rainy Lake eventually drains into the Lake of the Woods, but water level changes at these dams do not have an influence on water level in the Lake of the Woods.

Three of the large lakes in the Namakan complex, Lake Kabetogama, Namakan Lake and Sand Point Lake (described elsewhere [12]), and Rainy Lake were treated as ‘Impact’ lakes in this study. Water level fluctuations in Lake Kabetogama, Namakan Lake and Sand Point Lake are regulated by dams at Kettle and Squirrel Falls on Namakan Lake (Fig 1). The Namakan complex was the main focus of the 2000 rule curve changes, but we also treated Rainy Lake as impacted since there were subtle changes in water level management of Rainy Lake from the 1970 Rule Curves to the 2000 Rule Curves [11] and those changes and their secondary effects may have affected Walleye, Northern Pike, and Yellow Perch. The ‘Control’ lakes in this study are Vermilion Lake, which is similar geologically and ecologically to the Rainy-Namakan complex lakes, and Lake of the Woods, which is similar ecologically and at a similar latitude [17]. Appropriate data exists from some other large lakes of northern Minnesota (e.g., Cass Lake, Leech Lake), but these lakes occur within a very different geologic context [17]. Although the Rainy-Namakan complex drains into Lake of the Woods, it is a very small portion of the total watershed for that lake.

### Fish catch data

For all control and impacted lakes, standardized annual gill-net data was generally available for greater than 10 years before and after the 2000 rule curve change in the Rainy-Namakan complex. For Namakan Lake, data prior to 1993 are excluded due to an incompletely documented change in methodology. For Sand Point Lake, no data from 1992 were provided. Data were collected by the Minnesota Department of Natural Resources as part of their large lakes sampling program. The sampling methods have been described in detail elsewhere [18]. Some variation in sampling locations occurred through time [18], therefore only sampling locations that were sampled from 1990–2014 were included in this analysis. After examining the data, 3 species were selected that frequently occur in gillnet samples, and have ecological and management significance: Walleye, Northern Pike and Yellow Perch.

Adult fish populations likely do not respond immediately to changes in water level management and also are not usually able to be captured by this sampling gear until age 2–3, therefore we included a 5-year lag between the implementation of the 2000 Rule Curve and the years we included as representing the Post-2000 Rule Curve sample. The Pre-2000 period was from 1990–1999 and the Post-2005 from 2005–2014.

### Statistical methods

In the standard BACI configuration, each sampling event is characterized by two factors: Before/After (BA) to indicate whether the sample is from before or after the impact and Control/Impact (CI) to indicate whether the sample is from an impacted site or a control site. A BACI analysis suggests an impact has occurred when the interactions between these factors is non-zero: That is when the magnitude of the BA factor is different for the control samples than the impact samples. We used a simple Bayesian approach (as in [19], Chapter 3), to estimate the mean differences in CPUE between the pre-2000 and post-2005 periods in both control and impact lakes. This difference in means is the before-after effect. When the difference in means varies between the control and impact lakes, the BACI framework suggests the impact has altered the fish community.

Mean catch per gill net set was estimated using counts from individual gill-net sets from the Pre-2000 and Post-2005 time periods. Because gillnet counts are always non-negative integers, we were interested in using a distribution that is restricted to non-negative integers such as the Poisson distribution. However, the Poisson distribution assumes the population is randomly dispersed among sampling locations and times, which is unlikely to be the for many fish species. For example, Yellow Perch often move in groups and Northern Pike tend to occur in particular habitats [20]. Therefore, we used a hierarchical model to incorporate the possibility that fish occur in ‘clumps’ (as in [19]), by modeling among-sampling event variation in mean catch as a log-normal distribution and the sampling variation as a Poisson distribution. We refer to this hierarchical model of mean catch per gill net set as the Poisson-lognormal estimate. The Poisson-lognormal estimate will have larger estimates of variation for species with a more clumped spatial distribution. These models were implemented in R [21] using the rjags package [22]. Example code is provided in the statistical appendix (S1).

## Results

### Changes in fish community CPUE

In the before-after (BA), control-impact (CI) framework, the primary question is whether the BA effect differs between the control and impact lakes. In this study, the difference in mean catch per gill-net set (CPUE) Pre-2000 and Post-2005 is the BA effect, so if the differences are greater or less in the control lakes than in the impact lakes this would be evidence for the ‘impact’ altering fish community CPUE.

**Fig 2:**
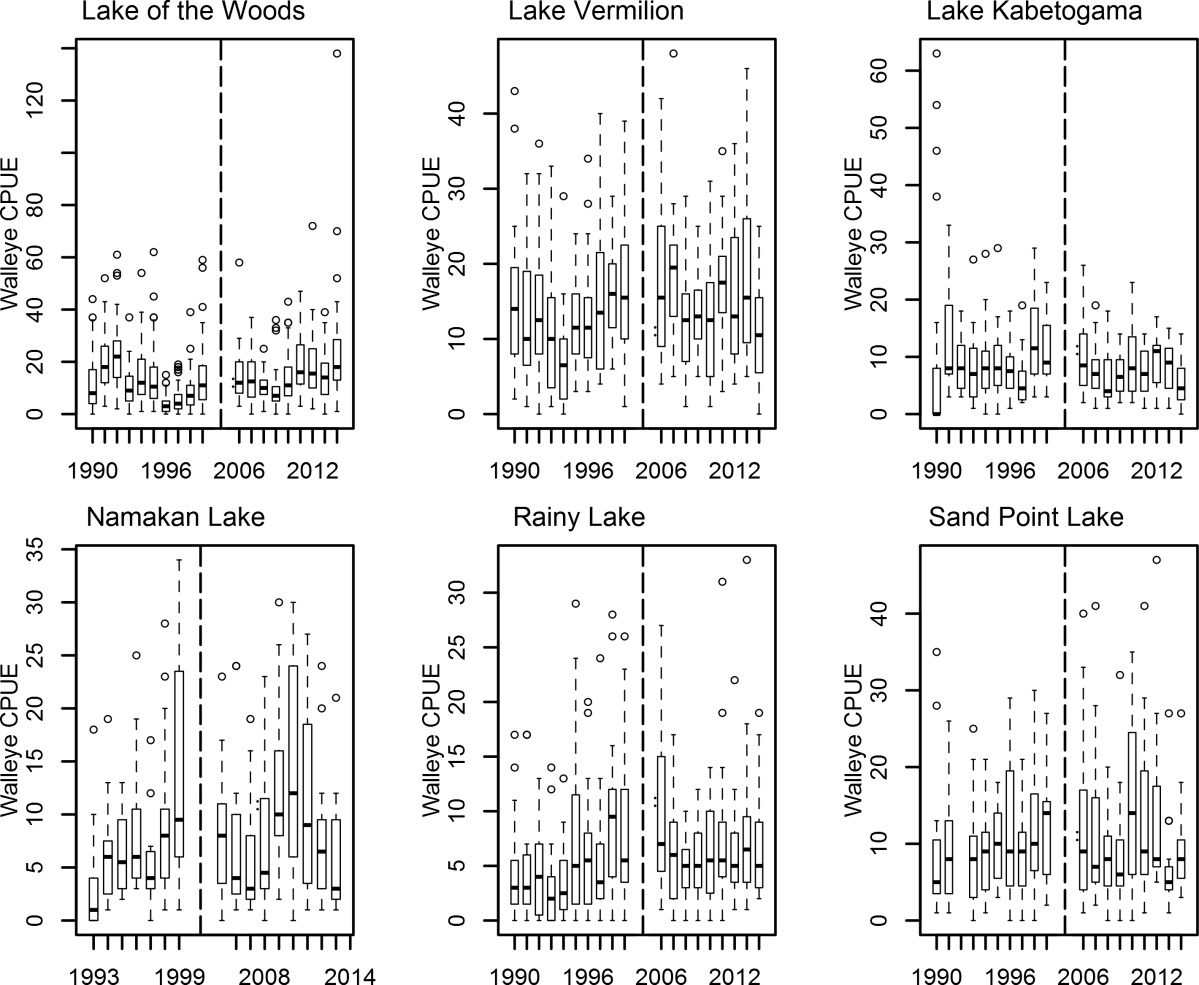
Box and whisker plots showing median Walleye catch per gillnet set (CPUE) in study lakes from 1990–1999 and from 2006–2014. Boxes encompass the first and third quartile. The lines (whiskers) show the largest or smallest observation that falls within 1.5 times the box size. Observations that fall outside the lines are shown individually. Lake Kabetogama, Namakan Lake, Rainy Lake and Sand Point Lake were all impacted by a water level management change that occurred in 2000. Lake of the Woods and Lake Vermilion were unaffected by the 2000 water level management change. The vertical dashed line separates the Pre-2000 and Post-2005 years.

For Walleye, both control lakes (Lake of the Woods and Lake Vermilion) experienced increases in Walleye CPUE that were distinguishable from zero (Table 1, Fig 2). Only one of the four impacted lakes experienced an increase in Walleye CPUE that was distinguishable from zero (Rainy Lake, Table 1). However, the 95% credible interval of the difference in Lake Vermilion overlapped the 95% credible interval of the differences in all of the impact lakes (Table 1). On the other hand, Lake of the Woods and the three most directly impacted lakes (Lake Kabetogama, Namakan Lake and Sand Point Lake) had differences that were distinct (i.e., no overlap of 95% credible intervals; Table 1). Therefore the choice of control lake affects our conclusions.

**Table 1:** The difference in mean catch per gill-net set before Pre-2000 and Post-2005 six northern Minnesota lakes. Means from the Pre-2000 and Post-2005 period are calculated using a hierarchical model that accounts for non-random spatial dispersion of fish (Poisson-lognormal). Differences with 95% credible intervals that do not overlap zero are highlighted with **bold** text. Positive differences indicate an increase in catch per gill-net set.

Lake	Species	Estimated difference
Lake of the Woods^C^	Walleye	**4.24**_**(3.12 to 5.34)**_
Lake Vermilion^C^		**2.35**_**(0.68 to 4.01)**_
Lake Kabetogama		-0.13_(-1.32 to 0.97)_
Namakan Lake		0.88_(-0.37 to 2.11)_
Rainy Lake		**2.09**_**(1.3 to 2.87)**_
Sand Point Lake		0.58_(-1.08 to 2.31)_
Lake of the Woods^C^	Northern Pike	**0.94**_**(0.76 to 1.11)**_
Lake Vermilion^C^		**-0.36**_**(-0.6 to -0.13)**_
Lake Kabetogama		**0.66**_**(0.26 to 1.06)**_
Namakan Lake		**0.86**_**(0.46 to 1.27)**_
Rainy Lake		**0.48**_**(0.17 to 0.81)**_
Sand Point Lake		**0.94**_**(0.45 to 1.44)**_
Lake of the Woods^C^	Yellow Perch	-0.51_(-1.63 to 0.62)_
Lake Vermilion^C^		**-5.85**_**(-9.5 to -2.26)**_
Lake Kabetogama		**4.23**_**(2.93 to 5.57)**_
Namakan Lake		-0.10_(-0.77 to 0.58)_
Rainy Lake		0.68_(-0.13 to 1.62)_
Sand Point Lake		**-0.86**_**(-1.52 to -0.27)**_

Lakes labeled with a ^C^ (control) were not influenced by the change in water level management.

**Fig 3:**
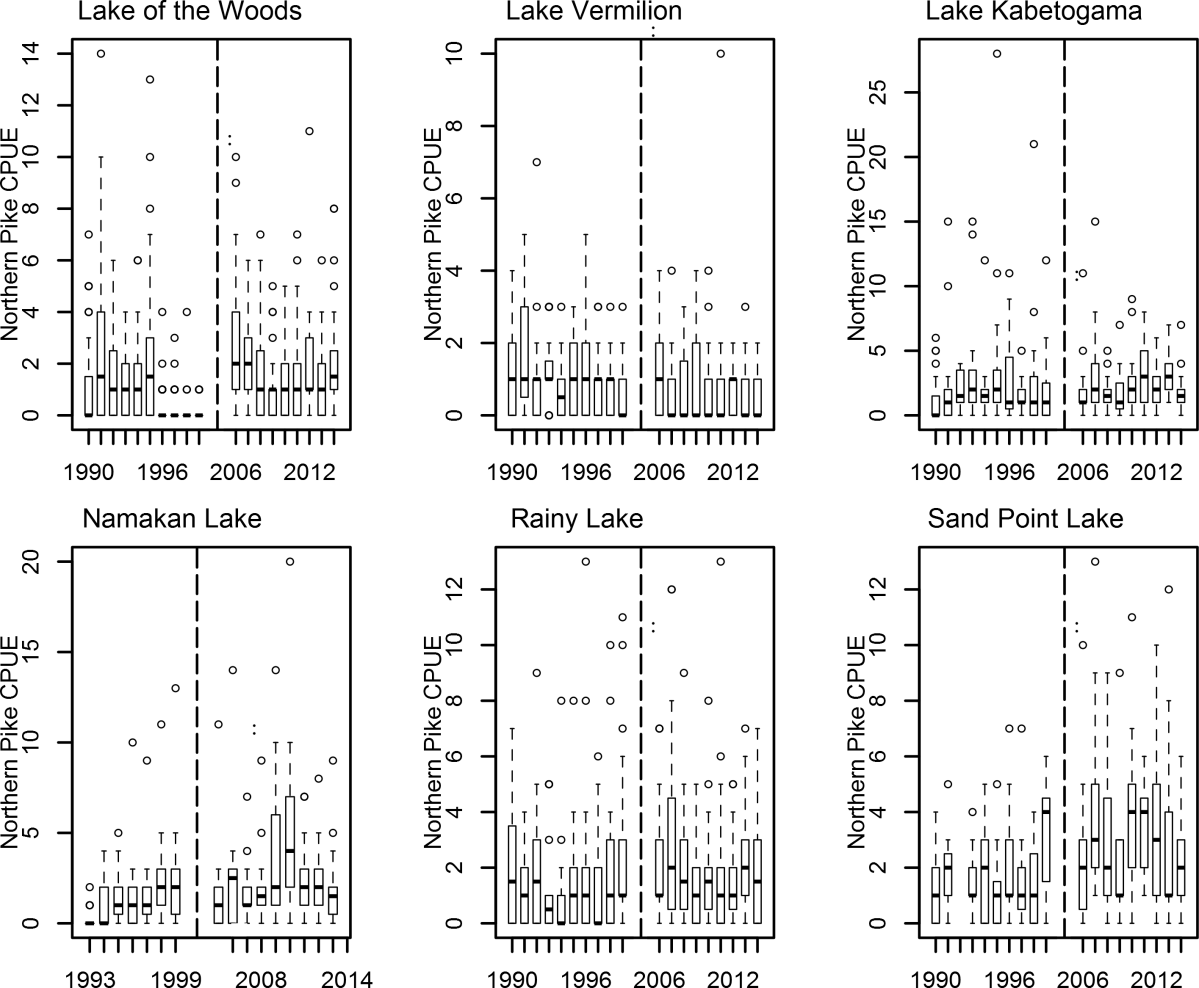
Box and whisker plots showing median Northern Pike catch per gillnet set (CPUE) in study lakes from 1990–1999 and from 2006–2014. Boxes encompass the first and third quartile. The lines (whiskers) show the largest or smallest observation that falls within 1.5 times the box size. Observations that fall outside the lines are shown individually. Lake Kabetogama, Namakan Lake, Rainy Lake and Sand Point Lake were all impacted by a water level management change that occurred in 2000. Lake of the Woods and Lake Vermilion were unaffected by the 2000 water level management change. The vertical dashed line separates the Pre-2000 and Post-2005 years.

For Northern Pike, CPUE decreased in Lake Vermilion (Fig 3), with a 95% credible interval that did not overlap with any other lake (Table 1). However, all five of the other lakes had mean differences that had overlapping 95% credible intervals (Table 1) and all were positive. Again, this indicates that the choice of control lake affects our conclusions.

**Fig 4:**
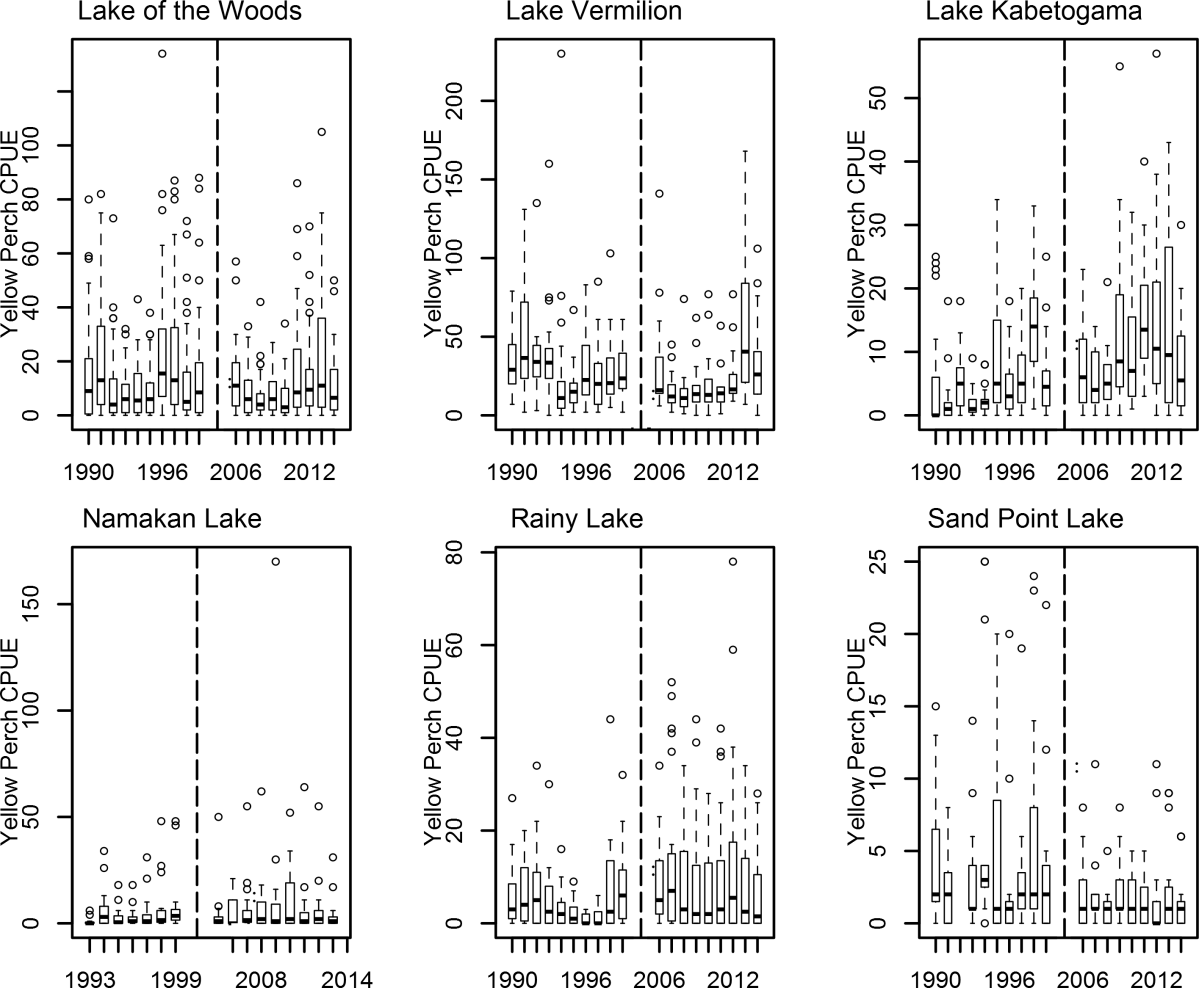
Box and whisker plots showing median Yellow Perch catch per gillnet set (CPUE) in study lakes from 1990–1999 and from 2006–2014. Boxes encompass the first and third quartile. The lines (whiskers) show the largest or smallest observation that falls within 1.5 times the box size. Observations that fall outside the lines are shown individually. Lake Kabetogama, Namakan Lake, Rainy Lake and Sand Point Lake were all impacted by a water level management change that occurred in 2000. Lake of the Woods and Lake Vermilion were unaffected by the 2000 water level management change. The vertical dashed line separates the Pre-2000 and Post-2005 years.

For Yellow Perch, in Lake of the Woods the mean difference between the before and after periods was slightly negative, but had a 95% credible interval that overlapped zero (Table 1, Fig 4). For Lake Vermilion (the other control lake), there was a substantial decline in Yellow Perch CPUE (Table 1). As a result, three of the impact lakes had differences in CPUE that overlapped with at least 1 control lake (Table 1). However, Lake Kabetogama had a substantial increase in Yellow Perch CPUE that did not overlap with either control lake.

## Discussion

The primary objective of this study was to identify whether a change in water level management in the Rainy-Namakan complex was associated with changes in an index of Walleye, Northern Pike and Yellow Perch abundance. When specific mechanisms that might drive changes in fish abundance are well understood and amenable to modeling, the specific effects of water level fluctuations can be evaluated using specific statistical models [15]. However, water level fluctuations have an array of ecological effects, and it is difficult or impossible to model the aggregate effect of these changes. In the before-after, control-impact analytical framework (BACI), evidence for the impact of a known change can be evaluated without mechanistic modeling [23]. In the BACI analysis completed here, the impact was the change in water level management that occurred in 2000 and altered water levels in Lake Kabetogama, Namakan Lake, Sand Point Lake, and Rainy Lake [11]. This analysis suggests that Yellow Perch CPUE was positively impacted by this change in water level management in Lake Kabetogama.

### Choice of control lake

If we only had access to data from one control lake, our analysis would lead to very different conclusions about the change in water level management. For example, if we had data from Lake Vermilion but not Lake of the Woods, we might conclude that Northern Pike and Yellow Perch were strongly influenced by the change in water level management. If we had data from only Lake of the Woods, we might conclude that Walleye were negatively impacted by this water level management change. The two lakes we used as controls here are appropriate due to a shared geologic and ecological setting, but there is no clear *a priori* reason to treat one of these control lakes as the ‘preferred’ option.

In the case of BACI analysis, more control sites are better [24]. Potential control sites typically outnumber impact sites. Sampling many control sites allows for analysis of spatial variation in the temporal trends of the variables of interest [24]. Unfortunately, in the case of these large northern lakes, there are relatively few potential control sites and relatively little long-term data. Other large lakes for which data are available (e.g., Cass Lake, Lake Winnibigoshish, Leech Lake) occur further south, within distinct geological settings [17], more watershed development and somewhat different fish communities (although all three of these species occur widely).

### Identifying changes in the fish community caused by the 2000 Rule Curves

In this analysis, Northern Pike increased in all of the impact lakes after the implementation of the new water level management program. There are some clear hypotheses about why the previous water level management regime may have negatively impacted Northern Pike in particular (reviewed in [2,13,25]). However, increases also occurred in Lake of the Woods, which was not affected by these water level management changes. Underwood and others [26] stress the difficulty in ascribing causality to field-based observations such as those described here. Although statistical differences can be observed, the mechanistic cause of those differences can never be unambiguously ascribed to the impact of interest. Often researchers use experimental and field observations in concert to ascribe causality [27,28], but this is often not feasible for whole-system manipulations such as the one studied here.

Identifying impacts of the 2000 Rule Curves on fish communities is perhaps particularly difficult because of high variability in fish capture efficiency, relatively low sample size for mechanistic modeling and on-going changes to other factors that influence fish communities, including climate and fisheries management. The estimated changes in abundance (as inferred from CPUE) of Walleye, Northern Pike and Yellow Perch identified in Lake of Woods and Lake Vermilion suggest that this region is experiencing considerable shifts in fish populations independent of any water level management occurring in the Rainy-Namakan complex (see also [29]). As a result, it is difficult or impossible to have complete confidence in any conclusions about the cause behind particular changes in abundance in these lakes.

The analysis here suggests that the implementation of the 2000 Rule Curves is associated with increased abundance of Yellow Perch in Lake Kabetogama. Water level fluctuations have a variety of effects on aquatic ecosystems [1,30,31], and the cause of this change is not known. In a previous study, we found evidence that age-zero Yellow Perch were more abundant in Lake Kabetogama after the new water level management program was started, but models relating water level fluctuation to Yellow Perch production explained a very small amount of variation in annual Yellow Perch production [15]. Future efforts will be needed to identify the mechanisms causing Yellow Perch increases in Lake Kabetogama and determine whether these mechanisms were influenced by water level fluctuations.

## Supporting information

S1 File Statistical appendix. Example code that shows how mean differences were calculated.
(DOCX)

S2 File Data appendix. All data used in this paper.
(XLSX)
