# Supplementary material for: Before-after, control-impact analysis of evidence for the impacts of water level on Walleye, Northern Pike and Yellow Perch in lakes of the Rainy-Namakan complex (MN, USA and ON, CA)
Source: PLoS One. 2018 Jun 7;13(6):e0198612. doi: 10.1371/journal.pone.0198612 (PMC5991717; doi:10.1371/journal.pone.0198612)
Supplement: S1 File — Example code that shows how mean differences were calculated. (DOCX) [file pone.0198612.s001.docx]

Statistical Appendix.

Fish data were compiled from data requests to the Minnesota Department of Natural Resources using a combination of R Version 3.1.0 [1] and SAS (SAS Institute). All statistics were completed in R.

Our approach was based on the Before-After, Control-Impact study design (BACI). Because adult fish populations probably do not respond instantly to changes in water level management, we included a 5-year lag between the implementation of the 2000 Rule Curve and the years we included as representing the post-2000 Rule Curve sample. Therefore the Before period was from 1990-1999 and the After is from 2005-2014. Years 1990-1993 in Namakan Lake were excluded due to a known problem in data management (B. Vondra, pers. comm.) and data from 1992 in Sand Point Lake was not provided (possibly never collected). In this example, the mean differences are calculated for the pre-2000 (Before) and post-2005 (After) periods for Sand Point Lake Walleye population, first using the Poisson distribution and then using the PoissonPlus method. These methods are derived from McCarthy [2]. This was done for each Control (Lake of the Woods, Lake Vermilion) and Impact lake (Sand Point Lake, Lake Kabetogama, Rainy Lake, Namakan Lake). If the mean differences between the pre-2000 and post-2005 CPUEs differ between Control and Impact lakes, that would imply the impact may have influence fish CPUE.

Files referenced in this code are included as individual spreadsheets in the data appendix (S2 File).

library("plyr")

library("coda", lib.loc="C:/Program Files/R/R-3.1.0/library")

library(rjags)

#This is the model we are using to fit the data

modelString = "

model {

for (i in 1:N) #Pre-2000 rule curve

{

mean[i]~dlnorm(Pre,tau1) #Adding the possibility of density differences in sampling locations/times (see McCarthy p 72)

x[i] ~ dpois(mean[i])

}

Pre ~ dnorm (1, 1.0E-6) #Wide, uninformative priors

sd1~dunif(0,10)

tau1<-1/(sd1*sd1)

for (j in 1:M) #Post-2000 rule curve

{

mean2[j]~dlnorm(Post,tau2) #Adding the possibility of density differences in sampling locations/times (see McCarthy p 72)

y[j] ~ dpois(mean2[j])

}

Post ~ dnorm (1, 1.0E-6) #Wide, uninformative prior

sd2~dunif(0,10)

tau2<-1/(sd2*sd2)

PostMedian<-exp(Post)

PreMedian<-exp(Pre)

d <- PostMedian-PreMedian #positive indicates more after the 2000 Rule Curve

}

"

writeLines(modelString,con="Meansmodel.txt")

### Info for all the models

adaptSteps = 1000 # Number of steps to "tune" the samplers.

burnInSteps = 10000 # Number of steps to "burn-in" the samplers.

nChains = 3 # Number of chains to run.

numSavedSteps=10000 # Total number of steps in chains to save.

thinSteps=2 # Number of steps to "thin" (1=keep every step).

nIter = ceiling((numSavedSteps * thinSteps)/nChains) # Steps per chain.

SandPointWalleye <- read.csv(file="~/SandPointWalleyeCatch.csv")

#this just needs to point towards the relevant file

SandPointWalleye90s<- subset(SandPointWalleye, YEAR<2000)

SandPointWalleye00s<- subset(SandPointWalleye, YEAR>2000)

datalist<- list("N"= length(SandPointWalleye90s$Total),"x" = SandPointWalleye90s$Total,

"M"= length(SandPointWalleye00s$Total),"y"= SandPointWalleye00s$Total)

parameters = c( "PostMedian","PreMedian","d") # The parameter(s) to be monitored.

jagsWalleyeSandPointModel = jags.model( "Meansmodel.txt" , data=datalist , # inits=initsList ,

n.chains=nChains , n.adapt=adaptSteps )

update( jagsWalleyeSandPointModel , n.iter=burnInSteps )

WalleyeSandPointcodaSamples = coda.samples( jagsWalleyeSandPointModel , variable.names=parameters ,

n.iter=nIter , thin=thinSteps )

WalleyeSandPointModelDiag<-gelman.diag(WalleyeSandPointcodaSamples,confidence = 0.95, transform=FALSE, autoburnin=FALSE,multivariate=FALSE)

WalleyeSandPointModelDiag<- data.frame(window(WalleyeSandPointModelDiag)$psrf)

WalleyeSandPointModelDiag$Difference <-WalleyeSandPointModelDiag$Point.est.-1

#This extracts the data into a data frame

WalleyeSandPointResults<-data.frame(round(summary(window(WalleyeSandPointcodaSamples ))$quantiles[,c(3, 1, 5)], 2))

WalleyeSandPointResults$Variable<-"WalleyeSandPoint"

WalleyeSandPointResults$Parameters<-as.factor(rownames(WalleyeSandPointResults))

WalleyeSandPointResults$RDiff<-WalleyeSandPointModelDiag$Difference

WalleyeSandPointResults$ConvergenceCheck <- ifelse(WalleyeSandPointResults$RDiff>0.1,

c("NoConvergence"), c("Convergence"))

#In this case, all our models converged, but if one did not, we would increase the number of steps

References:

1. R Development Core Team. R: A language and environment for statistical computing. [Internet]. Vienna, Austria: R Foundation for Statistical Computing; 2014. Available: http://www.r-project.org

2. McCarthy M. Bayesian methods for ecology. New York, New York, USA: Cambridge University Press; 2007.

3. Kruschke JK. Doing Bayesian Data Analysis. Oxford, UK: Elsevier; 2011.
